# Research on India-China agriculture trade dynamics: A comparative advantage analysis

**DOI:** 10.1371/journal.pone.0294561

**Published:** 2023-11-30

**Authors:** Swaraj Shukla, Kamilla Sadykova, Jinjin Tian

**Affiliations:** International College, Central South University of Forestry and Technology, Changsha, China; National Technical University of Athens: Ethniko Metsobio Polytechneio, GREECE

## Abstract

This paper presents a comprehensive analysis of the agriculture trade dynamic between India and China from 2002–2021. It employed the Revealed Comparative Advantage (RCA) and Revealed Symmetric Comparative Advantage (RSCA) indices and various statistical analyses to assess the trade performance and provide an enhanced comprehension of the specialization pattern. The study has selected 45 agricultural commodities, categorized into seven sections according to the HS Nomenclature 2017. The findings revealed that India boasts a more diversified export portfolio compared to China, with a more significant number of agriculture chapters demonstrating a comparative advantage. Conversely, China’s export basket is more concentrated, featuring fewer chapters with a comparative advantage but higher values of RCA and RSCA. The paper also identified the prospective areas for agriculture-trade cooperation and collaboration between the two countries and put forward recommendations. This research aims to offer valuable insights for policymakers, researchers, and traders to enhance bilateral ties and mutual benefits in the agriculture sector.

## 1. Introduction

India and China were among the 23 original signatories of the General Agreements on Tariffs and Trade (GATT or GATT-47) on October 30^th^, 1947. However, China’s subsequent withdrawal from the GATT system in 1949 changed its economic trajectory [1]. On April 1^st^, 1950, India became the first non-socialist country to establish diplomatic relations with the People’s Republic of China [2]. In 1978, China commenced economic reforms that marked a significant shift from a centrally planned economy to a market-oriented economy. The Household Responsibility System, which granted farmers greater autonomy, significantly improved agricultural productivity. These reforms also contributed to China’s economic revival by ensuring food security and generating an excess labor force for urban industrialization. The simultaneous implementation of these reforms was pivotal in China’s history [3]. India’s economic reforms happened nearly twelve years later, in 1991, with the primary objective of fostering liberalization and modernization within the country’s economy. However, unlike China, agriculture reforms were not the central part of India’s 1991 economic reform. One of the reasons was that, unlike the industrial sector, despite being the most significant GDP contributor, the agriculture sector has historically lacked centralization and regulation in India since its independence [4]. At the same time, under the GATT-47 contract, India maintained its engagement and later became one of the original World Trade Organization (WTO) members established on January 1^st^, 1995. China’s accession to the WTO occurred nearly five years later, on December 11^th^, 2001, a milestone in its global trade integration [5]. Today, India and China stand as two of the most populous and fastest-growing economies, with significant roles in global trade and development. Furthermore, both countries are hailed as agrarian economies, as agriculture is one of the most substantial contributors to their respective GDPs. In the fiscal year 2020–2021, agriculture constituted 20% of India’s gross value-added and employed more than 50% of the workforce [6]. India and China are also two of the world’s top agriculture exporters, as demonstrated in Table 1 below.

**Table 1:** India and China’s total trade volume and percentage share in the world’s total trade volume (All values are in USD billion).

Year	World’s total trade volume	India’s total trade volume	India’s % share in world’s total trade volume	India’s YoY growth rate	China’s total trade volume	China’s % share in world’s total trade volume	China’s YoY growth rate
**2002**	13045.612	107.551	0.824	-	620.766	4.758	-
**2003**	15214.684	131.791	0.866	5%	850.988	5.593	18%
**2004**	18511.303	174.885	0.945	9%	1154.555	6.237	12%
**2005**	20985.267	241.214	1.149	22%	1421.906	6.776	9%
**2006**	24257.174	299.413	1.234	7%	1760.397	7.257	7%
**2007**	27951.864	364.543	1.304	6%	2176.175	7.785	7%
**2008**	32408.668	497.573	1.535	18%	2563.255	7.909	2%
**2009**	25070.909	443.167	1.768	15%	2207.202	8.804	11%
**2010**	30440.761	570.438	1.874	6%	2973.766	9.769	11%
**2011**	36514.974	763.886	2.092	12%	3641.783	9.973	2%
**2012**	36924.815	778.541	2.108	1%	3866.981	10.473	5%
**2013**	37727.46	802.657	2.128	1%	4158.999	11.024	5%
**2014**	37795.744	776.914	2.056	-3%	4301.528	11.381	3%
**2015**	32988.29	655.126	1.986	-3%	3953.032	11.983	5%
**2016**	32001.263	617.032	1.928	-3%	3685.558	11.517	-4%
**2017**	35355.245	738.417	2.089	8%	4107.164	11.617	1%
**2018**	39000.627	830.108	2.128	2%	4620.045	11.846	2%
**2019**	37852.081	802.134	2.119	0%	4578.492	12.096	2%
**2020**	35212.59	643.469	1.827	-14%	4658.666	13.23	9%
**2021**	44530.764	965.216	2.168	19%	6046.665	13.579	3%
			**AAGR**	**5.579%**		**AAGR**	**5.785%**
			**CAGR**	**5.219%**		**CAGR**	**5.675%**

Source: Author’s calculation based on the data sourced from UNCOMTRADE.

Furthermore, China has maintained its position as India’s leading trade partner (for export and import) over the last decade. Notably, China is also one of the largest markets for India’s agricultural products, further signifying its importance as a trade partner. Moreover, considering China’s prominent role as one of India’s leading trade partners in recent years, a trade imbalance exists where China enjoys a surplus while India is in deficit [7]. The agriculture sector significantly impacts several sectors of a country’s economy, including industry, services, transport, and tourism, due to its forward and backward linkage. Agriculture has the potential to contribute to the enhancement of social welfare through the mitigation of poverty, inequality, and malnutrition [8]. Agriculture is a crucial factor for ensuring both food security and economic growth (i.e., generating income, employment, trade facilitation, etc.). However, it faces potential challenges due to the conflicting demands for food prices. India and China are prominent agriculture producers and consumers, encountering similar concerns such as climate change, food security, and rural development. Therefore, both countries stand to gain advantages by sharing best practices, innovations, and technologies in the agriculture sector [9]. Despite the agriculture sector being one of the most significant drivers of the Indian and Chinese economies, a detailed assessment of the evolution and comparison of agriculture trade between the two countries remains elusive. While previous research by Batra A, Khan Z. (2005) [10] investigated the structure of the comparative advantage of India and China in the global market individually and in a comparative framework, there exists a notable research gap, especially the ones that focuses on the comparison of agriculture trade between the two countries. The limited number of studies examining the complex dynamics of agriculture trade between India and China has been intensified by the unavailability of data and the unreliability of sources. This research gap substantially hinders the possibility of comprehensive academic research in this field. Therefore, it is evident that there is a pivotal need for a comprehensive investigation that effectively addresses this research gap. In light of this context, the present study aims to conduct a thorough comparative analysis of agriculture trade dynamics between China and India and identify the products with comparative advantages, disadvantages, and future trade potential. April 1st, 2020, marked 70 years of diplomatic relations between India and China. Both countries currently share multiple global platforms, such as BRICS, SAARC, SCO, G20, WTO, etc., and serve as prominent trade allies, maintaining strong economic and bilateral ties. Therefore, a comprehensive research on the comparative advantage of agriculture trade dynamics between these two countries can help us identify the areas with potential for collaboration and specialization that can positively impact bilateral ties, improve trade imbalances and present future cooperation opportunities.

## 2. Literature review

### 2.1 A comprehensive review of comparative advantage in agriculture: Assessing Revealed Comparative Advantage (RCA) and Revealed Symmetric Comparative Advantage (RSCA) indices

In his book, *“On the Principles of Political Economy Taxation*,*”* first published in 1817, David Ricardo proposed the theory of comparative advantage, which elucidated the occurrences of international trade even when one country possesses superior efficiency in producing all goods compared to others. Since then, scholars have employed various economic indicators, such as trade balance and international market share, to analyze and quantify comparative advantage. In 1965, Bela Balassa put forward the theory of "Revealed Comparative Advantage." He proposed the RCA Index, which suggested that a country’s comparative advantage can be indicated by its trade performances in the sense that the commodity pattern of trade reflects relative costs and differences in non-price factors [11]. In 1998, Dalum B, Laursen K, Villumsen G. investigated the stability and development of OECD export specialization patterns using Balassa’s RCA Index and proposed the RSCA (Revealed Symmetric Comparative Advantage) Index to avoid the problems of zero values that can occur in logarithmic transformation [12].

Shuai C., Wang X [13] conducted an empirical analysis of the comparative advantage and complementarity of the Sino-US agriculture trade in terms of sixteen major agriculture products since 1997 using multiple quantitative methods (i.e., RCA, etc.). Sharma SK, Bugalya K. [14] assessed the competitiveness of India’s agriculture sector in terms of cotton production and export employing RCA, Bilateral RCA, and TCI. Narayan S, Bhattacharya P. [15] examined India’s relative export competitiveness in four agriculture commodities and its determinants over the period 1961–2012, using time-varying measures of competitiveness based on the RCA index. Zhang F. [16] analyzed the agricultural trade between China and Brazil using quantitative research methods (i.e., RCA, TCI, and TI). Tao Z. [17] employed RCA and other quantitative methodologies to analyze the agriculture trade between Thailand and China to determine the advantages and disadvantages of agricultural trade on a competitive and complementary scale. Zhou L, Tong G [18] examined and analyzed the trade competitiveness of agricultural products between China and the countries along the “Belt and Road” using descriptive statistics and visual analysis. Wang, L., Sun, T., & Cai, Z. [19] studied the dynamics of Chinese export comparative advantage from 2001 to 2020 using the RSCA index and various statistical methods. Zhang TT, Yang J. [20] conducted a network analysis of global agricultural trade from 1991 to 2021, using data from various sources and the QAP regression method.

The studies mentioned above highlight two notable points: the importance of RCA and RSCA indices in examining and assessing the comparative advantage of agriculture trade, trade trends, patterns, and relative efficiency in producing specific agriculture commodities. Moreover, RCA facilitates the assessment of trade specialization within the agricultural industry. It highlights the goods in which a nation exports a greater proportion of its total exports. At the same time, the RSCA index offers a symmetrical perspective of comparative advantage by considering both exports and imports. Therefore, these indices enable researchers to comprehend a better specialization pattern that reveals the field in which a nation demonstrates a clear comparative advantage. The second notable point is that several studies have examined the comparative advantage of agriculture trade involving India and China; however, only a few researchers have investigated the bilateral agriculture trade between India and China. Therefore, there is an extensive research gap concerning the comparative advantage of agriculture trade between the two countries. Therefore, by examining and integrating the findings from the existing research, this study aims to provide a contemporary and comprehensive understanding of similarities and differences in the structure of comparative advantage in agriculture trade between the two countries over time and the implications for trade patterns and market dynamics. Through conscientious analysis, this study aims to provide policymakers, researchers, and vice versa with valuable insight that can help them formulate strategies to enhance trade cooperation and capitalize on the comparative advantages in the agriculture trade between India and China.

### 2.2. An overview of India-China bilateral trade

#### 2.2.1. An analysis of India-China total bilateral trade

As illustrated in Table 2, in the year 2021, the total bilateral trade volume between India and China exhibited a 41% year-on-year growth, reflecting a substantial upsurge of 34% in comparison to the previous year, reaching a total of 125.323 billion USD, indicating strengthening economic interdependence of the two countries. AAGR and CAGR for the selected period 2002–2021 were respectively 21.387% and 19.144%, indicating an upward trajectory of bilateral trade. The descriptive statistics analysis showed that India’s average trade volume remains comparatively lower than China’s, indicating distinct trade patterns. China’s trade data demonstrates a higher degree of variability than India’s, while the relative variability of total bilateral trade between India and China was moderate. Moreover, the total bilateral trade data exhibits a nearly symmetrical distribution with a mesokurtic distribution pattern, indicating a state of equilibrium in the trade relationship, signifying mutual gains for both countries through trade. The calculated Pearson’s correlation (r = 0.833) indicates a moderately strong and significant linear relationship between the trade volumes of India and China. Furthermore, the analysis underscores that approximately 69.3% of India’s trade can be attributed to China’s trade volume, further highlighting the importance of bilateral trade in economic stability.

**Table 2:** India-China total bilateral trade & total bilateral agriculture trade (All values are in USD billion).

Year	India’s total trade volume with China	China’s total trade volume with India	Total bilateral trade volume	YoY growth of total bilateral trade volume	India’ total agriculture trade volume with China	China’s total agriculture trade volume with India	Total bilateral agriculture trade volume	YoY growth of total agri-culture volume
**2002**	1.611	2.883	4.494	-	0.226	0.442	0.668	-
**2003**	2.699	3.532	6.231	39%	0.317	0.386	0.703	5%
**2004**	4.371	6.132	10.503	69%	0.486	0.407	0.893	27%
**2005**	7.582	9.199	16.781	60%	0.895	0.477	1.372	54%
**2006**	8.989	14.870	23.859	42%	2.187	0.512	2.699	97%
**2007**	10.784	24.380	35.164	47%	2.672	0.679	3.351	24%
**2008**	11.700	31.965	43.665	24%	2.805	0.789	3.594	7%
**2009**	11.303	30.149	41.452	-5%	1.932	0.964	2.896	-19%
**2010**	19.976	41.410	61.386	48%	5.198	1.013	6.211	114%
**2011**	20.420	51.372	71.792	17%	7.281	1.382	8.663	39%
**2012**	18.839	48.456	67.295	-6%	7.945	1.358	9.303	7%
**2013**	19.711	48.996	68.707	2%	7.344	1.161	8.505	-9%
**2014**	15.744	54.781	70.525	3%	4.472	1.184	5.656	-33%
**2015**	10.760	58.719	69.479	-1%	2.258	1.036	3.294	-42%
**2016**	9.733	58.974	68.707	-1%	1.761	1.19	2.951	-10%
**2017**	13.493	68.723	82.216	20%	2.067	1.322	3.389	15%
**2018**	17.809	77.168	94.977	16%	3.072	0.99	4.062	20%
**2019**	20.041	75.379	95.420	0%	5.565	1.021	6.586	62%
**2020**	21.717	67.221	88.938	-7%	5.755	0.881	6.636	1%
**2021**	27.254	98.069	125.323	41%	8.233	0.967	9.2	39%
			**AAGR**	**21.387%**			**AAGR**	**20.944%**
			**CAGR**	**19.144%**			**CAGR**	**14.802%**

Source: Author’s calculation based on the data sourced from UNCOMTRADE.

#### 2.2.2. An analysis of India-China total bilateral agriculture trade

In 2021, the total bilateral agriculture trade volume between India and China reached an impressive 9.2 billion USD, exhibiting a 39% year-on-year (YoY) growth, a sustainable upswing compared to the previous year’s 1%. The average annual growth rate (AAGR) and compound annual growth rate (CAGR) for 2002–2021 were 20.444% and 14.802%, indicating evolving agriculture trade between the two countries, as shown in Table 2. The descriptive statistics analysis showed that agriculture trade volume exhibits higher variability and fluctuation than China. India and China’s total bilateral agriculture trade volume exhibit slightly elongated tails towards higher value, indicating significant trade patterns, possibly driven by specific agricultural sectors. Moreover, the overall distribution is slightly platykurtic, indicating thinner tails and less peakedness compared to a standard normal distribution, implying the existence of specific elements shaping the agriculture trade between the two countries. The Pearson correlation coefficient (r = 0.6653) indicated a statistically significant positive linear association between the agricultural trade volumes of India and China.

**Fig 1:**
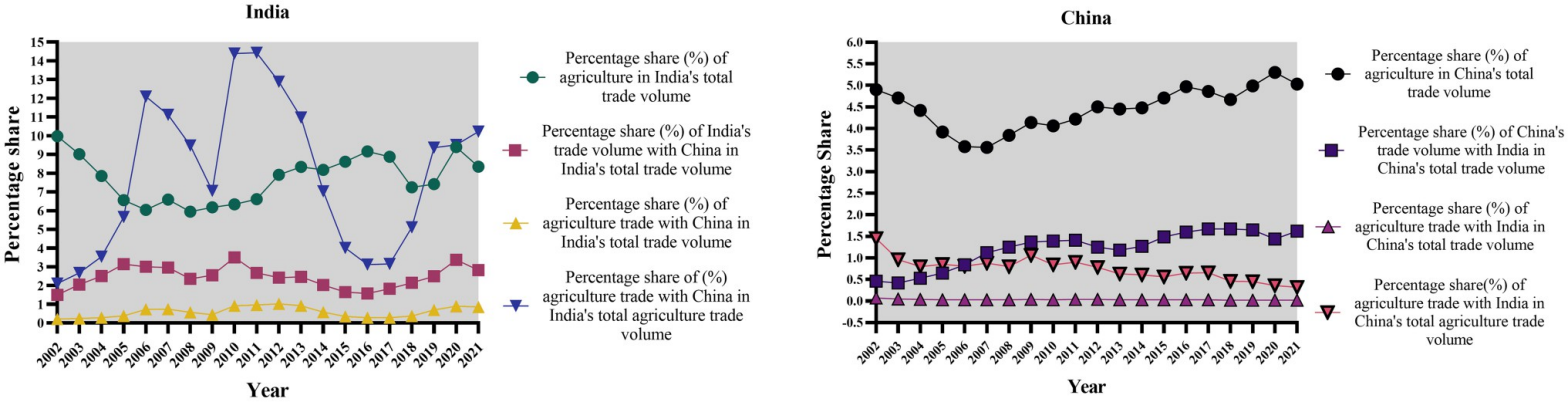
Comprehensive analysis of India and China’s agriculture trade shares. Source: Author’s creation based on the statistical analysis and data sourced from UNCOMTRADE.

Moreover, a significant portion, precisely 44.26%, of the variability in India’s agriculture trade volume can be attributed to the variability in China’s agriculture trade volume. Therefore, the correlation between agriculture trade between India and China is moderate and reliable. This observation emphasizes the significant impact of China’s trade activities on India’s trade dynamics, illustrating the interdependence of the two economies in the agricultural trade sector. Fig 1. below provides a comprehensive analysis of India and China’s agriculture trade shares, highlighting the bilateral trade dynamics.

**Fig 2:**
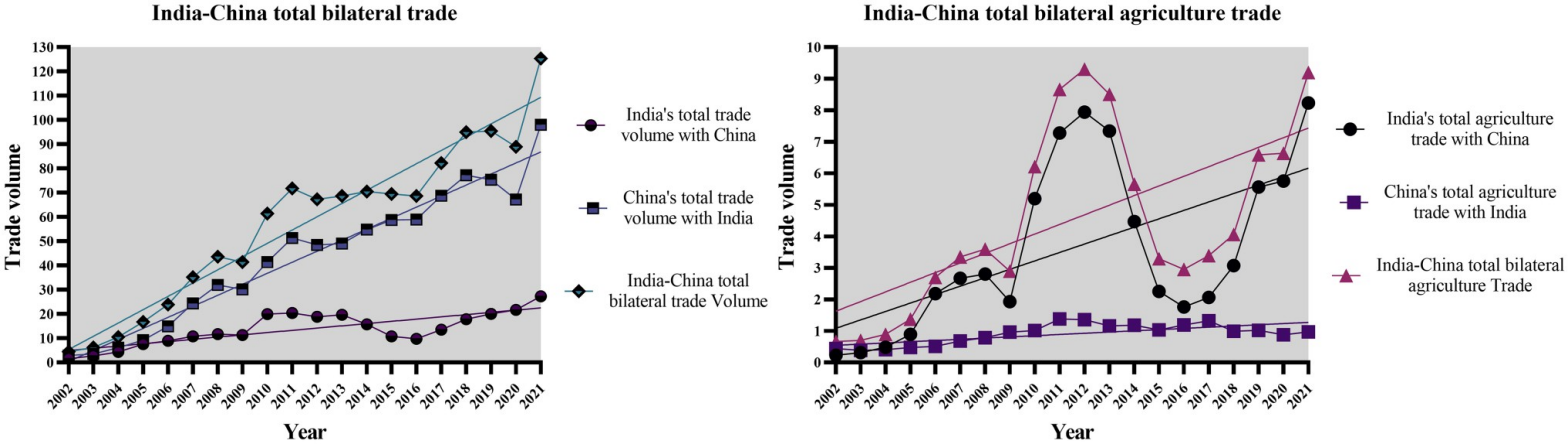
India-China total bilateral trade vs India-China total bilateral agriculture trade. Source: Author’s creation based on the statistical analysis and data sourced from UNCOMTRADE.

**Fig 3:**
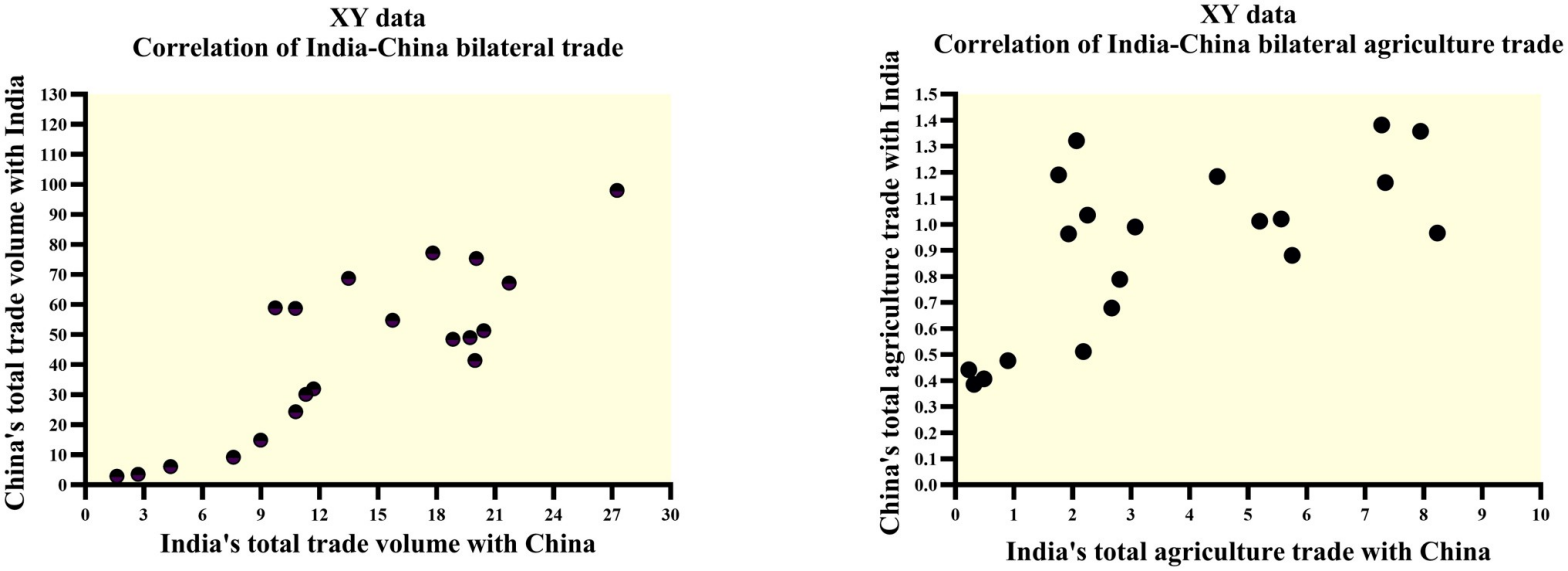
Correlation analysis of India-China total bilateral trade vs India-China total bilateral agriculture trade. Source: Author’s creation based on the statistical analysis and data sourced from UNCOMTRADE.

**Fig 4:**
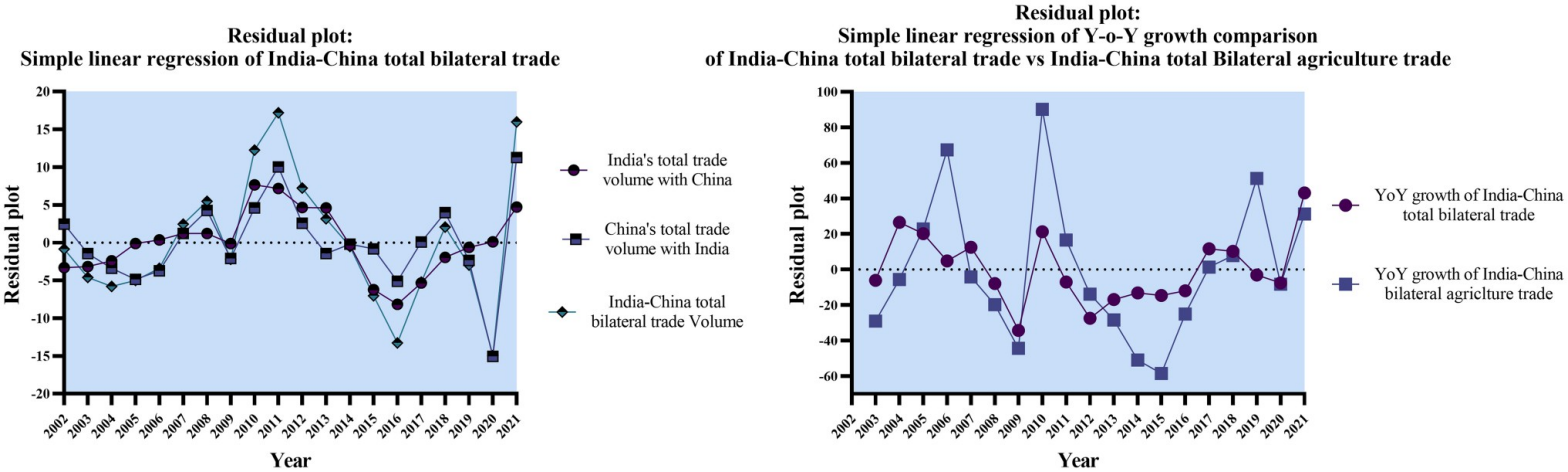
Simple linear regression of India-China total bilateral trade vs India-China total bilateral agriculture trade. Source: Author’s creation based on the statistical analysis and data sourced from UNCOMTRADE.

Figs 2–4 below provide us with a more profound and thorough assessment of the trade dynamics of India and China.

#### 2.2.3 Year-on-Year (Y-o-Y) growth comparison of India-China total bilateral trade volume and total bilateral agriculture trade volume

**Fig 5:**
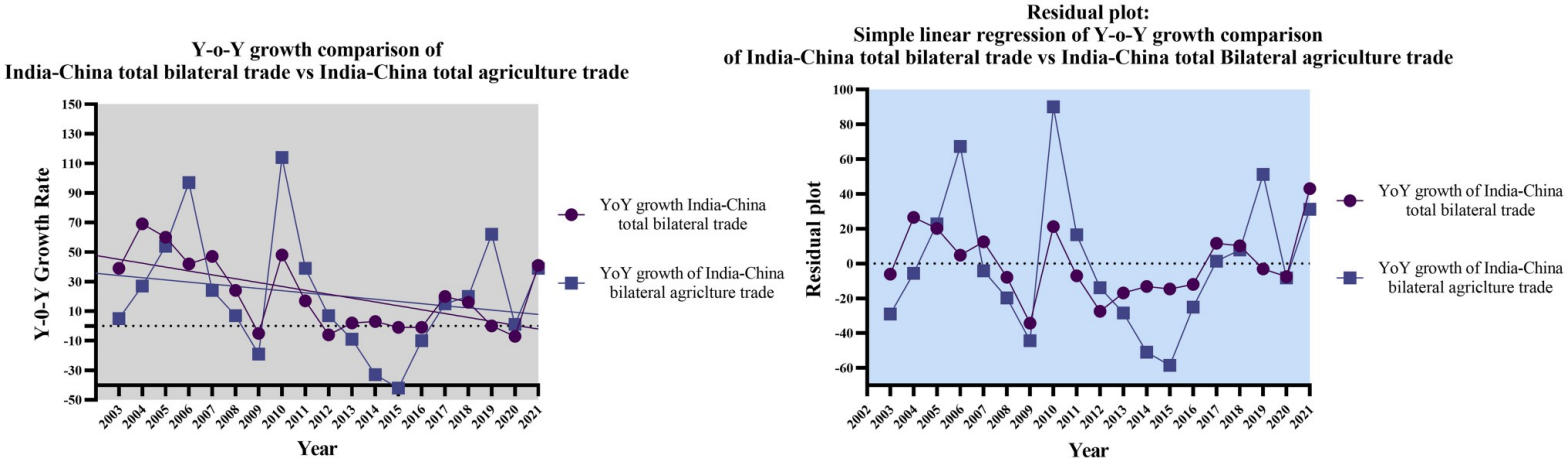
Year-on-Year growth comparison of India-China bilateral trade vs India-China bilateral agriculture trade. Source: Author’s creation based on the statistical analysis and data sourced from UNCOMTRADE.

The descriptive statistics analysis revealed that the total bilateral trade volume and total bilateral agriculture trade volume between India and China exhibit favorable growth rates, emphasizing the incremental nature of trade engagement between the two countries. However, the total bilateral agriculture trade volume showed higher relative variability, a more prominent right-skewed distribution (with higher skewness), and more substantial tails (with higher kurtosis) than the overall bilateral trade volume. The overall descriptive statistics findings indicate a likelihood of more significant heterogeneity and asymmetry in bilateral agriculture trade growth rates. The results could be attributed to various sector-specific elements, market trends, and regulatory compliances influencing the agriculture trade between India and China. The computed Pearson’s Correlation coefficient (r = 0.590) indicates a statistically significant moderate positive correlation between India-China Total Bilateral YoY Growth Rates and India-China Total Bilateral Agriculture YoY Growth rates. Furthermore, 34.8% of the variability in YoY Growth rates of total bilateral trade volume can be explained by the linear relationship with bilateral agriculture trade. Fig 5 below offers a graphical assessment of the interdependence between India-China total bilateral trade volume and total bilateral agriculture trade volume.

### 2.3. In-depth analysis of India and China’s agriculture trade dynamics

#### 2.3.1. India’s agriculture trade with China

In 2021, India’s total agriculture trade volume with China displayed a year-on-year growth of 43%, amounting to 8.232 billion USD. For the selected period of 2002–2021, the average annual growth rate (AAGR) and compound annual growth rate (CAGR) of India’s total agriculture trade with China were recorded as 32.199% and 20.831%, as displayed in Table 3. Descriptive statistics analysis revealed a relatively high level of variability in India’s agriculture imports and exports to China. The distribution is slightly skewed to the right. It has a lighter tail (platykurtic), suggesting fewer outliers than expected in a normal distribution, emphasizing the stability and predictability of agriculture trade patterns. Calculated Pearson’s Correlation coefficient (r-0.983) indicated a very strong positive linear relationship between India’s agriculture imports and exports to China, suggesting that as imports increase, exports tend to increase as well, and vice versa.

**Table 3:** India and China’s agriculture trade dynamics (All values are in USD billion).

Year	India’s agriculture import from China	India’s agriculture export to China	India’s total agriculture trade volume with China	YoY growth of India’s agriculture trade with China	China’s agriculture import from India	China’s agriculture export to India	China’s total agriculture trade volume with India	YoY growth of China’s agriculture trade with India
**2002**	0.079	0.147	0.226		0.212	0.23	0.442	
**2003**	0.132	0.185	0.317	40%	0.	0.197	0.386	-13%
**2004**	0.272	0.214	0.486	53%	0.196	0.211	0.407	5%
**2005**	0.398	0.497	0.895	84%	0.265	0.212	0.477	17%
**2006**	1.16	1.027	2.187	144%	0.289	0.223	0.512	7%
**2007**	1.292	1.38	2.672	22%	0.329	0.35	0.679	33%
**2008**	1.606	1.199	2.805	5%	0.38	0.409	0.789	16%
**2009**	0.933	0.999	1.932	-31%	0.483	0.481	0.964	22%
**2010**	2.536	2.661	5.197	169%	0.496	0.517	1.013	5%
**2011**	3.702	3.579	7.281	40%	0.836	0.546	1.382	36%
**2012**	4.11	3.835	7.945	9%	0.779	0.579	1.358	-2%
**2013**	3.294	4.05	7.344	-8%	0.564	0.597	1.161	-15%
**2014**	2.31	2.162	4.472	-39%	0.564	0.62	1.184	2%
**2015**	1.183	1.074	2.257	-50%	0.491	0.545	1.036	-13%
**2016**	0.817	0.944	1.761	-22%	0.576	0.614	1.19	15%
**2017**	0.998	1.069	2.067	17%	0.68	0.642	1.322	11%
**2018**	1.433	1.639	3.072	49%	0.492	0.498	0.99	-25%
**2019**	2.762	2.804	5.566	81%	0.554	0.467	1.021	3%
**2020**	2.709	3.047	5.756	3%	0.502	0.379	0.881	-14%
**2021**	4.217	4.015	8.232	43%	0.558	0.409	0.967	10%
			**AAGR**	**32.199%**			**AAGR**	**5.425%**
			**CAGR**	**20.831%**			**CAGR**	**4.207%**

Source: Author’s calculation based on the data sourced from UNCOMTRADE

#### 2.3.2. China’s agriculture trade with India

As shown in Table 3 2021, China’s total agriculture trade volume with India reached 0.967 billion USD in 2021, exhibiting a 10% year-on-year growth. For the selected period of 2002–2021, the average annual growth rate (AAGR) and compound annual growth rate (CAGR) for China’s agriculture trade with India were recorded as 5.425% and 4.207%, respectively, indicating a steady growth rate. The descriptive statistics analysis revealed a moderate variability in China’s agriculture imports and exports to India. The distribution for the import is roughly symmetric. In contrast, the distribution for the export has a lighter tail (platykurtic) than a normal distribution. It is somewhat skewed to the left, indicating fewer extreme values than would be the case in a normal distribution, indicating a certain level of stability and predictability in agriculture trade with India. Computed Pearson’s Correlation (r = 0.864) indicated a strong positive linear relationship between China’s agriculture imports and exports with India, signifying that while imports rise, exports often follow suit and vice versa.

#### 2.3.3. Year-on-Year growth comparison of India’s agriculture trade volume with China and China’s agriculture trade volume with India

**Fig 6:**
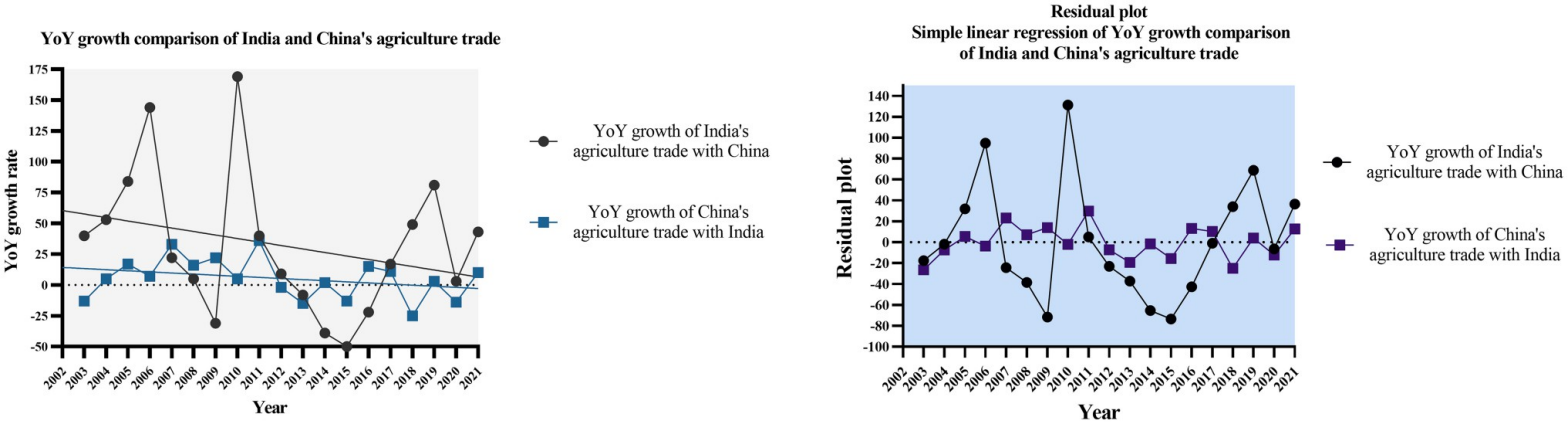
Year-on-Year growth comparison of agriculture trade volume of India and China. Source: Author’s creation based on the statistical analysis and data sourced from UNCOMTRADE.

The descriptive statistics analysis revealed that China’s year-on-year growth exhibits greater variability than India’s, indicating more fluctuations in yearly growth rates for the selected period. However, India’s year-on-year growth distribution is more positively skewed than China’s. China’s growth distribution has somewhat lighter tails than a normal distribution, and India’s growth distribution has slightly heavier tails than a normal distribution, indicating substantial expansions over 20 years. Fig 6 below provides a comprehensive visual representation of growth comparisons and the simple linear relationship between the year-on-year growth rate of India and China.

**Fig 7:**
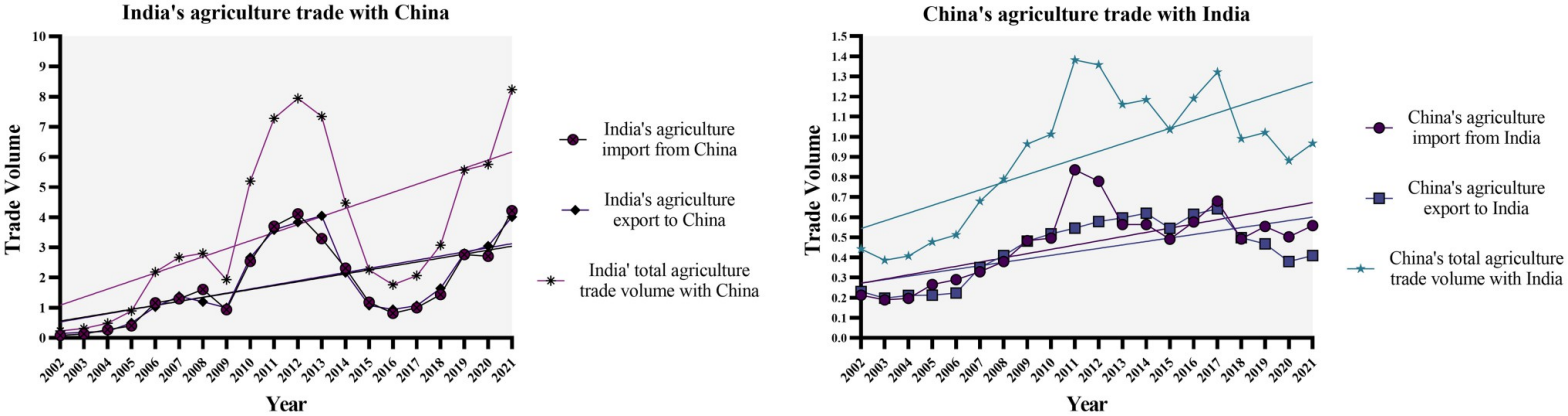
India’s agriculture trade with China vs China’s agriculture trade with India. Source: Author’s creation based on the statistical analysis and data sourced from UNCOMTRADE.

**Fig 8:**
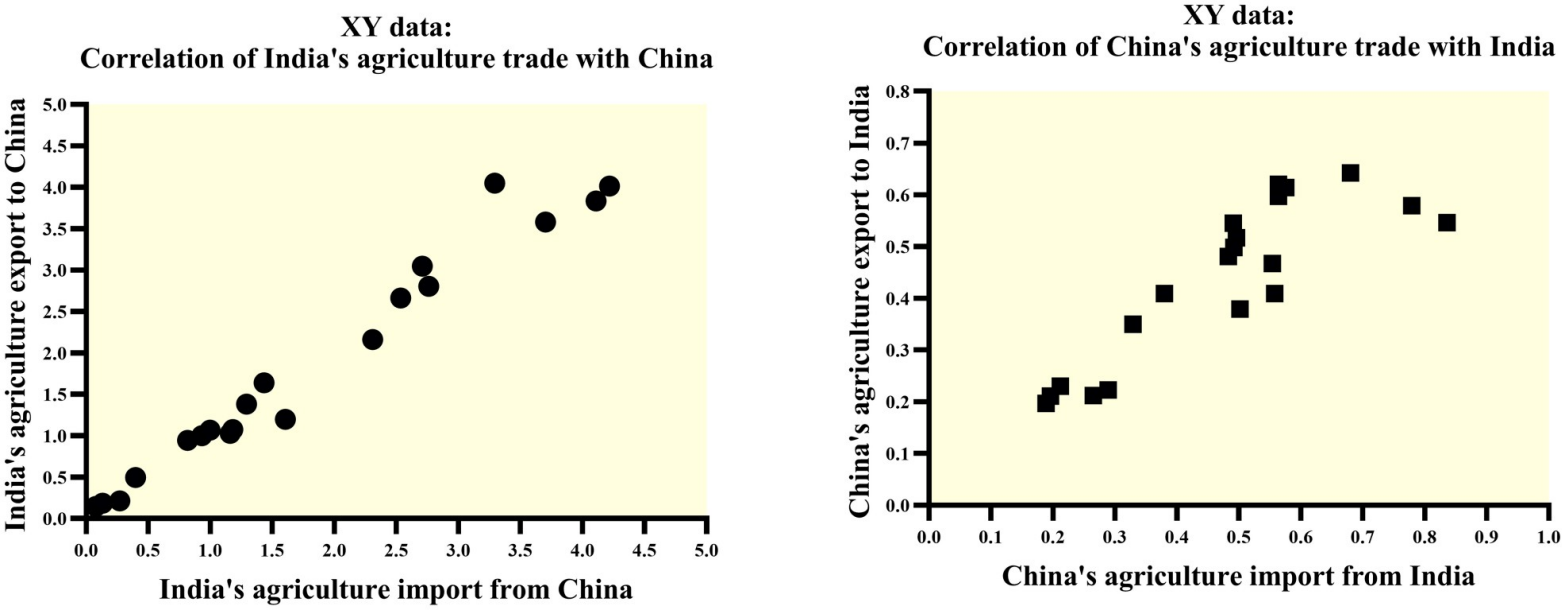
Correlation analysis of India’s agriculture trade with China vs China’s agriculture trade with India. Source: Author’s creation based on the statistical analysis and data sourced from UNCOMTRADE.

**Fig 9:**
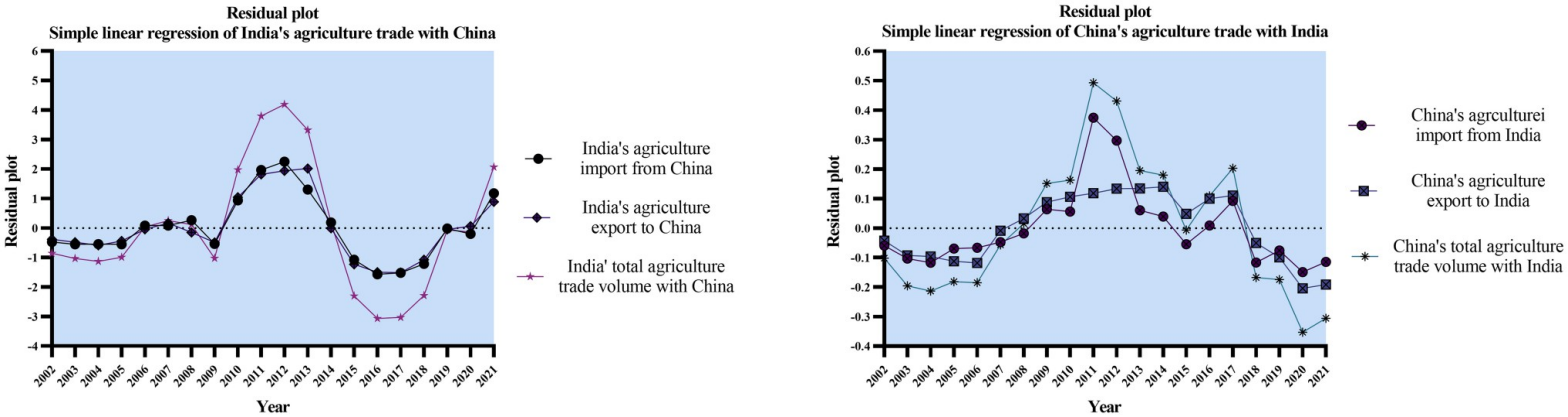
Simple linear regression of India’s agriculture trade with China vs China’s agriculture trade with India. Source: Author’s creation based on the statistical analysis and data sourced from UNCOMTRADE.

After conducting a thorough statistical analysis, it is evident that consistent growth rates, balanced distributions, substantial correlation, and linear relationships characterize the agricultural trade between China and India. These findings highlight the resilience and mutual benefits of the trade partnership between the two countries in the agricultural domain. Furthermore, Figs 7–9 below further accentuates the India- China agriculture trade, the correlation between agriculture imports and exports, and the simple linear regression of the agriculture trade between the two countries.

## 3. Data source and methodology

### 3.1. Data source and classification

In accordance with the definition of agriculture products by WTO, FAO, and UNCTAD, this study focuses on 45 agriculture commodities based on their significance in international trade. The study has chosen 20 years (2002–2021) of analysis as it provides comprehension of long-term trade patterns, trends, and policy changes. By analyzing the data over two decades, the study aims to comprehensively understand the dynamics and evolution of agriculture trade between India and China. The study has adopted the internationally recognized Harmonized System (HS) classification to ensure consistency and comparability in examining the trade data. At the two-digit level, Chapters HS01-HS24 have been included, while at the four-digit level, the following chapters are included: HS33, HS35, HS41, HS43, HS50, HS51, HS52, and HS53. Furthermore, the selected commodities have been classified into seven sections according to the HS Nomenclature 2017 [21] to facilitate data analysis and interpretation, as shown in Table 4 below. The data source utilized for this study is a combination of UNCOMTRADE and ITC TRADEMAP. By drawing trade data from multiple sources and using a systematic classification approach, this study ensures a robust and comprehensive analysis of the comparative advantage of agriculture trade between India and China.

**Table 4:** HS Nomenclature 2017.

Sections	Chapters & Subchapters
**SECTION I** **LIVE ANIMALS; ANIMAL PRODUCTS**	01-Live animals. 02-Meat and edible meat offal. 03-Fish and crustaceans, molluscs and other aquatic invertebrates. 04-Dairy produce; birds’ eggs; natural honey; edible products of animal origin, not elsewhere specified or included. 05-Products of animal origin, not elsewhere specified or included.
**SECTION II** **VEGETABLE PRODUCTS**	06-Live trees and other plants; bulbs, roots and the like; cut flowers and ornamental foliage. 07-Edible vegetables and certain roots and tubers. 08-Edible fruit and nuts; peel of citrus fruit or melons. 09-Coffee, tea, maté and spices. 10-Cereals. 11-Products of the milling industry; malt; starches; inulin; wheat gluten. 12-Oil seeds and oleaginous fruits; miscellaneous grains, seeds and fruit; industrial or medicinal plants; straw and fodder. 13-Lac; gums, resins and other vegetable saps and extracts. 14-Vegetable plaiting materials; vegetable products not elsewhere specified or included.
**SECTION III** **ANIMAL OR VEGETABLE FATS AND OILS AND THEIR CLEAVAGE**	15-Animal or vegetable fats and oils and their cleavage products; prepared edible fats; animal or vegetable waxes.
**SECTION IV** **PREPARED FOODSTUFFS; BEVERAGES, SPIRITS AND VINEGAR; TOBACCO AND MANUFACTURED TOBACCO SUBSTITUTES**	16-Preparations of meat, of fish or of crustaceans, molluscs or other aquatic invertebrates. 17-Sugars and sugar confectionery. 18-Cocoa and cocoa preparations. 19-Preparations of cereals, flour, starch or milk; pastrycooks’ products. 20-Preparations of vegetables, fruit, nuts or other parts of plants. 21-Miscellaneous edible preparations. 22-Beverages, spirits and vinegar. 23-Residues and waste from the food industries; prepared animal fodder. 24-Tobacco and manufactured tobacco substitutes.
**SECTION VI** **PRODUCTS OF THE CHEMICAL OR ALLIED INDUSTRIES**	**33-Essential oils and resinoids; perfumery, cosmetic or toilet preparations**. 3301-Essential oils, whether or not terpeneless, incl. concretes and absolutes; resinoids; extracted oleoresins; concentrates of essential oils in fats, fixed oils, waxes or the like, obtained by enfleurage or maceration; terpenic by-products of the deterpenation of essential oils; aqueous distillates and aqueous solutions of essential oils **35-Albuminoidal substances; modified starches; glues; enzymes**. 3501-Casein, caseinates and other casein derivatives; casein glues (excl. those packaged as glue for retail sale and weighing net < = 1 kg) 3502-Albumins, incl. concentrates of two or more whey proteins containing by weight > 80% whey proteins, calculated on the dry matter, albuminates and other albumin derivatives 3503-Gelatin, whether or not in square or rectangular sheets, whether or not surface-worked or coloured, and gelatin derivatives; isinglass; other glues of animal origin (excluding those packaged as glue for retail sale and weighing net < = 1 kg, and casein glues of heading 3501 3504-Peptones and their derivatives; other protein substances and their derivatives, n.e.s.; hide powder, whether or not chromed (excluding organic or inorganic compounds of mercury whether or not chemically defined) 3505-Dextrins and other modified starches, e.g. pregelatinised or esterified starches; glues based on starches, dextrins or other modified starches (excl. those put up for retail sale and weighing net < = 1 kg)
**SECTION VIII** **RAW HIDES AND SKINS, LEATHER, FURSKINS AND ARTICLES THEREOF; SADDLERY AND HARNESS; TRAVEL GOODS, HANDBAGS AND SIMILAR CONTAINERS; ARTICLES OF ANIMAL GUT (OTHER THAN SILK-WORM GUT)**	**41-Raw hides and skins (other than furskins) and leather.** 4101-Raw hides and skins of bovine "incl. buffalo" or equine animals, fresh, or salted, dried, limed, pickled or otherwise preserved, whether or not dehaired or split (excl. tanned, parchment-dressed or further prepared) 4102-Raw skins of sheep or lambs, fresh, or salted, dried, limed, pickled or otherwise preserved, whether or not dehaired or split (excl. those with wool on, fleeces of Astrakhan, Caracul, Persian, Broadtail or similar lambs, or of Indian, Chinese, Mongolian or Tibetan lambs and tanned, parchment-dressed or further prepared) 4103-Other raw hides and skins, fresh, or salted, dried, limed, pickled or otherwise preserved, whether or not dehaired or split (excl. those of bovine animals, equine animals, sheep and lambs, those with wool on and those of goats or kids from Yemen, Mongolia or Tibet and tanned, parchment-dressed or further prepared) **43-Furskins and artificial fur; manufactures thereof.** 4301-Raw furskins, incl. heads, tails, paws and other pieces or cuttings suitable for use in furriery (excl. raw hides and skins of heading 4101, 4102 or 4103)
**SECTION XI** **TEXTILES AND TEXTILE ARTICLES**	**50-Silk** 5001-Silkworm cocoons suitable for reeling 5002-Raw silk "non-thrown" 5003-Silk waste, incl. cocoons unsuitable for reeling, yarn waste and garnetted stock 5004-Silk yarn (excluding that spun from silk waste and that put up for retail sale) 5005-Yarn spun from silk waste (excluding that put up for retail sale) 5006-Silk yarn and yarn spun from silk waste, put up for retail sale; silkworm gut 5007-Woven fabrics of silk or of silk waste **51 Wool, fine or coarse animal hair; horsehair yarn and woven fabric** 5101-Wool, neither carded nor combed 5102-Fine or coarse animal hair, neither carded nor combed (excl. wool, hair and bristles used in the manufacture of brooms and brushes, and horsehair from the mane or tail 5103-Waste of wool or of fine or coarse animal hair, incl. yarn waste (excl. garnetted stock, waste of hair and bristles used in the manufacture of brooms and brushes, and of horsehair from the mane or tail) **52 Cotton** 5201-Cotton, neither carded nor combed 5202-Cotton waste, incl. yarn waste and garnetted stock 5203-Cotton, carded or combed **53-Other vegetable textile fibres; paper yarn and woven fabrics of paper yarn** 5301-Flax, raw or processed, but not spun; flax tow and waste, incl. yarn waste and garnetted stock. 5302-True hemp "Cannabis sativa L.", raw or processed, but not spun; tow and waste of true hemp, incl. yarn waste and garnetted stock

### 3.2. Methodology

#### 3.2.1. Balassa’s Revealed Comparative Advantage index (BRCA) 1965

The principle of comparative advantage is derived from the *Ricardian Trade Theory*, which postulates that relative productivity differences govern the trade pattern among countries. In 1965, Bela Balassa proposed the Revealed Comparative Advantage (RCA) index [22]. He asserted its significance in measuring comparative advantage as it utilizes available information on a nation’s trade performance (i.e., export shares, export-import ratios) in regard to various commodity categories. RCA assesses the degree to which a country’s export structure deviates from the expected trade patterns based on size. Furthermore, several prominent scholars, including Hillman AL. and Bowen H. P., have presented their interpretations of RCA. Bowen HP. [23] investigated the properties of the RCA index and found that the index can be used to identify a country’s comparative advantage and disadvantage in international trade. Ballance R, Pogany J, Forstner H. [24] Yeats AJ. [25], and Marchese S, Nadal De Simone F. [26] have analyzed the properties of various RCA indexes purported to approximate actual comparative advantage. French S. [27] investigated the usefulness of measures of revealed comparative advantage (RCA) in academic and policy analyses. RCA assesses the degree to which a country’s export structure deviates from the expected trade pattern based on size. Furthermore, RCA’s focus on revealed patterns, comparative assessment, dynamic analysis, and transparency makes it a competent index. Therefore, to analyze the comparative advantage of agriculture trade and the competitiveness of agriculture products between India and China, this study has adopted Balassa’s (1965) Revealed Comparative Advantage (BRCA) Index. The BRCA metric can be mathematically defined as follows-

$$BRCA_{i\dot{j}}^{k}=\left(X_{i\dot{j}}^{k}/X_{i\dot{j}}^{t}\right)/\left(X_{i_{w}}^{k}/X_{i_{w}}^{t}\right)$$ (1)

The numerator represents the percentage share of commodity k in the export of country i, while the denominator represents the percentage share of commodity k of country i in the world’s export. Where ${BRCA}_{i\dot{j}}^{k}$ is Balassa’s Revealed Comparative Advantage Index. $X_{i\dot{j}}^{k}$ and **$X_{ij}^{t}$** respectively refers to the export value of commodity k from country i to j and the total export value of country i to j for all the commodities. Similarly, $X_{i_{w}}^{k}\;and\;X_{iw}^{t}$ refers to the export value of commodity k from country i to the world and the total export value of country i to the world. The numerical value of Balassa’s RCA index ranges from 0 to infinity ($0\leq {BRCA}_{i\dot{j}}^{k}\leq \infty$), with 1 being the break-even point. That is, if ${BRCA}_{i\dot{j}}^{k}\leq 1$ it represents that commodity ***k*** in the country ***i*** has a relative disadvantage or lower comparative advantage. Conversely, ${BRCA}_{i\dot{j}}^{k}\geq 1$ means that the commodity ***k*** in country ***i*** has Revealed comparative advantage. ${BRCA}_{i\dot{j}}^{k}\geq 2.5$ means the commodity ***k*** in country ***i*** has a strong comparative advantage.

#### 3.2.2. Revealed Symmetric Comparative Advantage (RSCA) index

Balassa’s Revealed Comparative Advantage (BRCA) Index measures a nation’s relative export of specific products compared to the world average, allowing researchers to identify commodities in a nation with a competitive edge. It highlights potential export opportunities and formulation of trade strategies, but Hillman AL. [28] and Yeats AJ. [25] posit that the BRCA index needs to include the ordinal and cardinal properties. Furthermore, BRCA’s bias signifies a strong comparative advantage for commodities that comprise only a small market share of the world’s export. Dalum B, Laursen K, Villumsen G. [29] noted that the BRCA index has an inherent risk of normality because it takes between zero and infinity with a (weighted) average of 1.0, which can violate the assumption of normality of the error term in regression analysis and not produce reliable t-statistics. To alleviate this problem, they proposed using the RSCA index, which falls between +1.0 and -1.0 and avoids the problem of zero values that can occur in logarithmic transformation. Laursen K. [30] further analyzed BRCA and posited that RSCA is a better index than BRCA because it is symmetric around its natural value, making it the best comparative advantage measure. Furthermore, RSCA can be defined in the case of zero exports from a sector.

Therefore, RSCA allows for a more comprehensive assessment of a nation’s export performance and apprehend the strength and weaknesses in agriculture commodities. Furthermore, RSCA compliments the BRCA index by identifying sectors where both nations’ export performance is weaker than their respective overall average, which enables researchers and policymakers to identify the areas of improvement and formulate strategies to resolve potential challenges in the agriculture trade between China and India. This study has adopted the RSCA index proposed by Dalum B, Laursen K, Villumsen G. in 1998. The mathematical equation is as follows-

$$RSCA_{ij}=\frac{RCA_{ij}-1}{RCA_{ij}+1}$$ (2)

Where ***RSCA***_***ij***_ is Revealed Symmetric Comparative Advantage index for product ***j*** from country ***i***. The numerical value of RSCA index ranges from -1 to +1 **(-1≤*RCA***_***ij***_**≤+1).** When the ***RSCA***_***ij***_ index of a country **i** is positive, product **j** is said to have a comparative advantage. Conversely, if the ***RSCA***_***ij***_ index of country **i** is negative, product **j** is said to have a comparative disadvantage.

## 4. Result analysis and discussion of comparative advantage of agriculture trade between India and China

### 4.1. Results analysis of comparative advantage of agriculture trade between India and China

Based on the descriptive statistics of the calculated RCA and RSCA results of 45 agriculture commodities, divided into seven sections for the selected period of 2002–2021, in India and China, the study has the following findings:

a.) RCA Findings: As shown in Table 5, India exhibits moderate comparative advantage across all sections, with an average RCA value ranging from 0.734–3.69, with a mean of 1.81, indicating a relatively higher comparative advantage for India compared to China’s corresponding range of 0.586–3.15, with a mean of 1.12. Notably, for India, Section III (with the highest mean RCA = 5.19) exhibited strong comparative conversely, Section IV (lowest mean RCA value of 0.279) showed a comparative disadvantage. As for China, Section XI (highest mean value of 4.91) exhibited a comparative advantage, while Section I (with the lowest mean value of 0.105) revealed a comparative disadvantage. The standard deviation of India’s average RCA is 0.809, indicating moderate variability in RCA values, relatively higher than China’s corresponding value of 0.623. Regarding dispersion, section III for India and section XI for China showed the highest dispersion. Notably, the coefficient of variation for India’s average RCA was 44.70%, lower than China’s 55.70%, indicating stable variability in India’s RCA values. Regarding the distribution of the RCA values on average across all sections, India’s RCA distribution is moderately skewed to the right (skewness = 0.481). In contrast, China’s RCA distribution is more significantly right-skewed (skewness = 2.08), suggesting that both countries exhibit higher RCA value concentration; China’s distribution is more significant, indicating a narrower set of highly advantageous agriculture products.

**Table 5:** Descriptive statistics of India and China’s RCA results.

**Descriptive Statistics of India’s RCA**	**Section I**	**Section II**	**Section III**	**Section IV**	**Section VI**	**Section VIII**	**Section XI**	**Average RCA**
Number of values	20	20	20	20	20	20	20	20
Minimum	0.515	0.371	0.867	0.096	0.165	0	1.14	0.734
Maximum	2.7	1.92	9.34	0.769	0.972	4.96	7.49	3.69
Range	2.19	1.55	8.47	0.673	0.807	4.96	6.35	2.95
Mean	1.58	1.05	5.19	0.279	0.432	0.751	3.39	1.81
Std. Deviation	0.692	0.502	2.76	0.172	0.217	1.13	1.76	0.809
Std. Error of Mean	0.155	0.112	0.618	0.0385	0.0486	0.253	0.393	0.181
Coefficient of variation	43.80%	48.00%	53.30%	61.60%	50.40%	151%	51.80%	44.70%
Skewness	0.0503	0.307	-0.217	1.19	0.979	3.07	1.07	0.481
Kurtosis	-1.34	-1.14	-1.58	1.96	0.408	10.6	0.654	-0.247
**Descriptive Statistics of China’s RCA**	**Section I**	**Section II**	**Section III**	**Section IV**	**Section VI**	**Section VIII**	**Section XI**	**Average RCA**
Number of values	20	20	20	20	20	20	20	20
Minimum	0.007	0.26	0.155	0.068	0.502	0	1.92	0.586
Maximum	0.547	1.34	2.61	0.405	2.68	5.76	17.2	3.15
Range	0.54	1.08	2.45	0.337	2.18	5.76	15.3	2.56
Mean	0.105	0.498	0.476	0.207	1.03	0.597	4.91	1.12
Std. Deviation	0.15	0.257	0.549	0.0695	0.552	1.37	3.71	0.623
Std. Error of Mean	0.0335	0.0574	0.123	0.0155	0.123	0.307	0.829	0.139
Coefficient of variation	143%	51.60%	115%	33.60%	53.40%	230%	75.50%	55.70%
Skewness	2.19	2.04	3.43	1.27	1.9	3.18	2.35	2.08
Kurtosis	4.21	5.3	13.1	3.27	3.75	11.2	6.07	5.21

Source: Statistical Analysis based on the author’s RCA and RSCA calculations of India for 2002–2021.

b.) RSCA Findings: As shown in Table 6 below, RSCA results highlight that India (mean RSCA value -0.358) and China (mean RSCA value -0.531) indicate comparative disadvantage across all sections. However, India’s marginal higher average RSCA implies that India has a relatively better trade performance overall. Furthermore, Section III is the best-performing sector exhibiting comparative advantage with the highest mean RCA value for both India and China. Conversely, Section IV, with the lowest mean, exhibited a comparative disadvantage for both countries. Regarding dispersion, India (Std. Deviation = 0.087) and China (Std. Deviation = 0.072) have moderate variability in their RSCA values, as indicated by their standard deviations. Section XI has the highest volatility for both countries, indicating the most fluctuation in comparative advantage. On the other hand, section IV for India and Section II for China exhibited the lowest volatility, and the same is accurate in terms of variability relative to the mean or the coefficient of variation. Furthermore, regarding data asymmetry, India (skewness -0.79) has a negative value, indicating its RSCA distribution is skewed to the left. In contrast, China (skewness 1.19) has a positive skewness, indicating its RSCA distribution is skewed to the right. Section III has the highest negative skewness. Similarly, section XI has the highest positive skewness for India and China. In terms of tailedness, India (kurtosis = 1.44) and China (kurtosis = 1.35) have peaked distribution with heavier tails than the normal distribution, as indicated by their positive kurtosis values; however, India’s RSCA distribution is slightly stronger than that of China’s. Section III has the highest kurtosis value of India and China, indicating concentrated comparative advantage, while section IV has the lowest kurtosis values for both countries, indicating concentrated comparative disadvantage.

**Table 6:** Descriptive statistics of India and China’s RSCA result.

**Descriptive Statistics of India’s RSCA**	**Section I**	**Section II**	**Section III**	**Section IV**	**Section VI**	**Section VIII**	**Section XI**	**Average RSCA**
Number of values	20	20	20	20	20	20	20	20
Minimum	-0.575	-0.615	-0.071	-0.843	-0.82	-1	-0.528	-0.588
Maximum	-0.215	-0.226	0.806	-0.595	-0.493	0.132	0.236	-0.211
Range	0.36	0.389	0.877	0.248	0.327	1.13	0.764	0.377
Mean	-0.405	-0.449	0.578	-0.733	-0.677	-0.609	-0.211	-0.358
Std. Deviation	0.0825	0.117	0.248	0.0762	0.0893	0.295	0.208	0.087
Std. Error of Mean	0.0185	0.0261	0.0555	0.017	0.02	0.0659	0.0465	0.0195
Coefficient of variation	20.40%	26.00%	42.90%	10.40%	13.20%	48.40%	98.50%	24.30%
Skewness	-0.173	0.205	-1.18	0.124	0.108	0.66	0.722	-0.79
Kurtosis	0.719	-1.11	0.678	-1.26	-0.515	0.594	0.133	1.44
**Descriptive Statistics of China’s RSCA**	**Section I**	**Section II**	**Section III**	**Section IV**	**Section VI**	**Section VIII**	**Section XI**	**Average RSCA**
Number of values	20	20	20	20	20	20	20	20
Minimum	-0.717	-0.649	-0.731	-0.832	-0.644	-1	-0.546	-0.635
Maximum	-0.206	-0.233	0.446	-0.455	0.101	-0.521	0.065	-0.343
Range	0.511	0.416	1.18	0.377	0.745	0.479	0.611	0.292
Mean	-0.574	-0.5	-0.452	-0.659	-0.346	-0.871	-0.316	-0.531
Std. Deviation	0.146	0.105	0.292	0.0886	0.19	0.19	0.161	0.0724
Std. Error of Mean	0.0326	0.0236	0.0652	0.0198	0.0426	0.0424	0.0359	0.0162
Coefficient of variation	25.40%	21.10%	64.60%	13.40%	55.10%	21.80%	50.80%	13.60%
Skewness	1.78	0.616	1.83	0.903	0.45	1.03	0.85	1.19
Kurtosis	2.49	0.55	3.73	1.25	0.277	-0.849	0.19	1.35

Source: Statistical analysis based on the author’s RCA and RSCA calculations of China for 2002–2021

**Fig 10:**
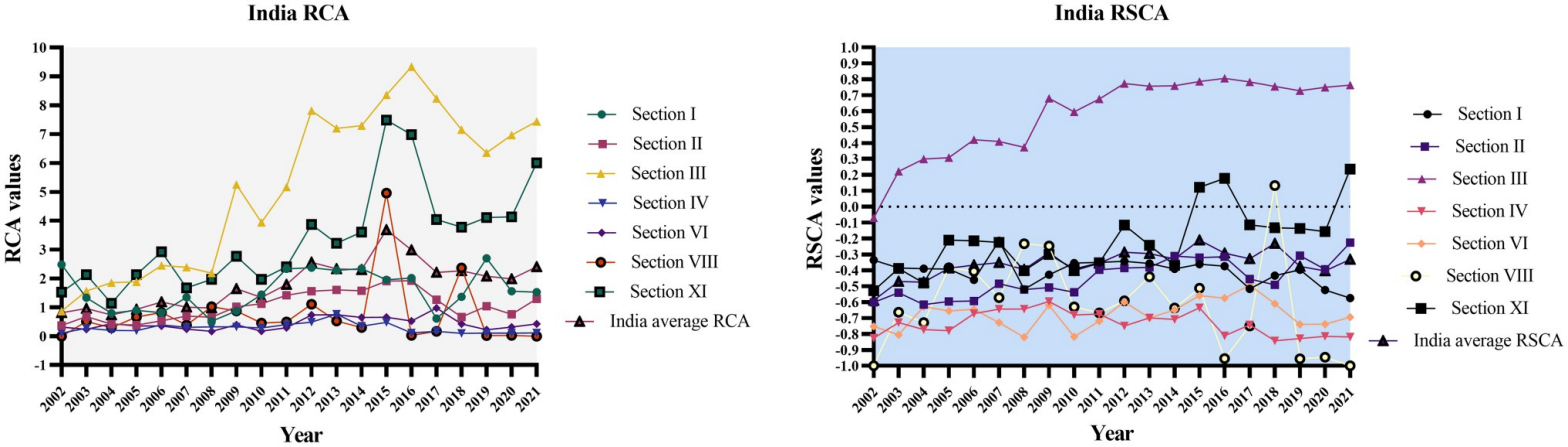
RCA and RSCA results of India’s agriculture trade (2002–2021). Source: Author’s creation based on the statistical analysis and data sourced from UNCOMTRADE.

**Fig 11:**
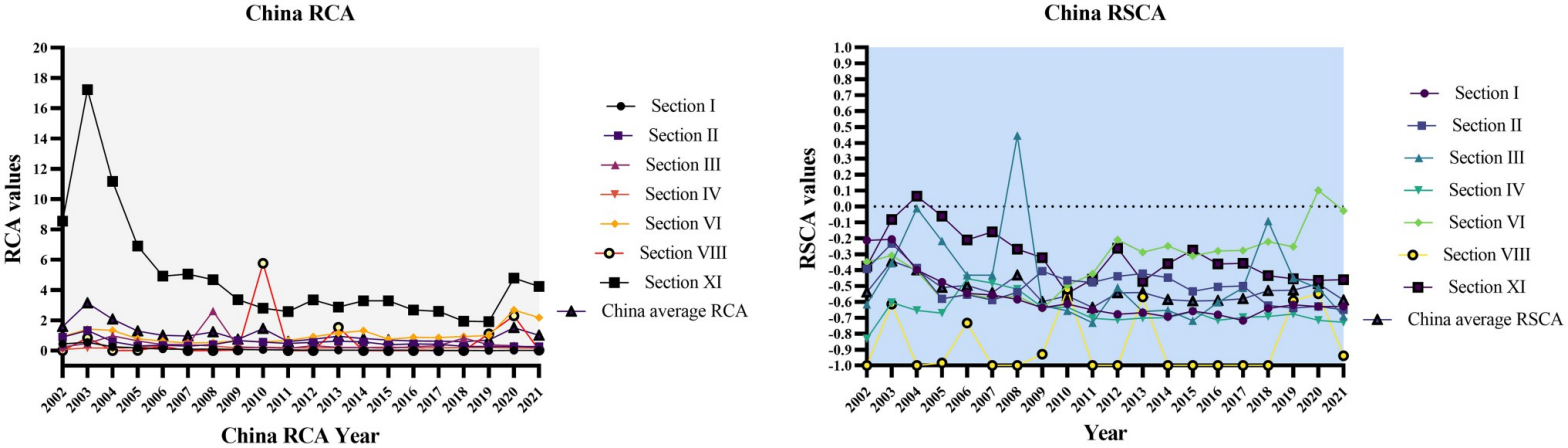
RCA and RSCA results of China’s agriculture trade (2002–2021). Source: Author’s creation based on the statistical analysis and data sourced from UNCOMTRADE.

The graphical representation of India and China’s RCA and RSCA for the chosen timeframe of 2002–2021 is depicted in Figs 10 and 11 below.

### 4.2. Discussions and trade development potential

Based on the findings derived from the RCA and RSCA analyses, the study has elucidated that India has a comparative advantage in six agriculture chapters. In comparison China has a comparative advantage in five agriculture chapters. Conversely, India has a comparative disadvantage in 14 agriculture chapters, while China has a comparative disadvantage in 24 agriculture chapters, as exhibited in the Table 7 below.

**Table 7:** Agricultural chapters demonstrating comparative advantage and comparative disadvantage in India and China.

Category	India	China
**Comparative Advantage**	03, 05, 14, 15, 13, 52	33, 35, 41, 50, 53
**Comparative Disadvantage**	01, 02, 04, 07, 08, 10, 16, 17, 18, 19, 20, 21, 22, 24, 43	01, 02, 03, 04, 06, 07, 08 09 10, 11, 12, 14, 15, 16, 17, 18, 19, 20, 21, 22, 23, 24, 51

Source: Author’s compilation derived from the RCA and RSCA analysis of India and China.

**Fig 12:**
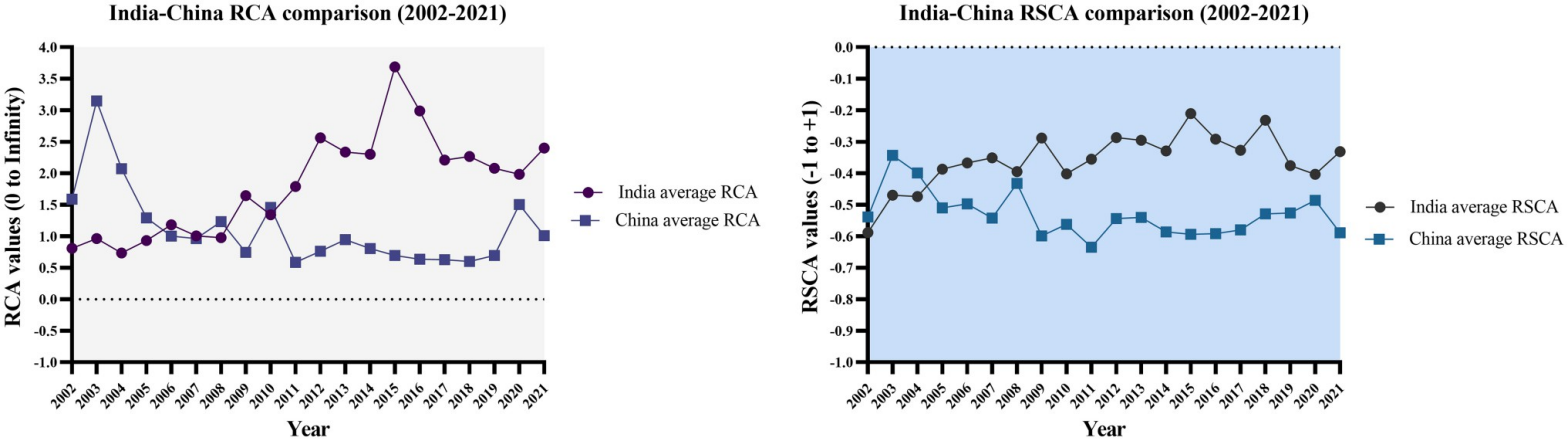
Comprehensive assessment of comparative advantage of agriculture trade between India and China (2002–2021). Source: Author’s creation based on the statistical analysis and data sourced from UNCOMTRADE.

Furthermore, it is evident that India and China exhibit mutual strengths and complementarity in some agriculture chapters (i.e., chapters HS13 and HS33), indicating a strong comparative advantage. It’s an indication of their abundant labor and land resources, as well as their emerging capital investment and technological innovation. Conversely, both countries face challenges in various agriculture chapters (i.e., 01, 02, 04, 07, 08, 10, 16, 17, 19, 20, 21, 22, 24). These sectors indicate India and China’s limitations in animal husbandry, food safety, agriculture industry development, and product quality. However, India’s overall higher RCA and RSCA results indicate that India has a more diverse agriculture portfolio with distinctive strengths than China. Fig 12 below illustrates the average RCA and RSCA values for the selected twenty-year periods.

India and China both exhibit mutual complementary strengths in various agriculture sectors (i.e., fisheries, oils, and textiles), which ensures demand fulfillment in both countries through bilateral trade. However, both countries face challenges in live animal and meat production, indicating a potential area for cooperation and mutual growth. India’s constraints in chemical and pharmaceutical products and China’s challenges in cocoa and food residues create opportunities for knowledge sharing and collaborative ventures. Likewise, in the leather industry, India excels in specific processes but encounters challenges in certain leather products, mirroring China’s difficulties in certain leather and fur product industries. It presents an opportunity for both countries to collaborate and enhance their capabilities in this sector. However, trade barriers in the form of tariffs, non-tariff measures, standards, and regulations need to be addressed through policy harmonization, increased investment, and improved trade logistics. India and China hold substantial potential for expanding bilateral agriculture trade, leveraging their vast and growing markets, diversified product portfolios, and complementary strengths. Therefore, by capitalizing on their strengths and addressing their challenges through cooperation and knowledge exchange, both countries can enhance their agricultural positions in the global agriculture trade network.

## 5. Conclusion and recommendations

### 5.1. Conclusion

The comprehensive analysis of the comparative advantage of agriculture trade between India and China has highlighted that India and China exhibit varying patterns of specialization and trade within the agriculture sector, signifying both competition and complementarity in their trade relationship. India boasts a more diversified export portfolio compared to China, with a greater number of agriculture chapters demonstrating comparative advantage. Conversely, China’s export basket is more concentrated, featuring fewer chapters with a comparative advantage but higher values of RCA and RSCA. The study has observed substantial growth and diversification in the agriculture trade of both countries over the last two decades. Notably, there is a robust positive correlation between the agriculture trade volumes of India and China, indicating interdependence and mutual benefits. Furthermore, the dynamics of agriculture trade are influenced by a multitude of external factors, such as trade policies, tariffs, non-tariff barriers, exchange rates, distance, and transportation costs. Therefore, future studies should utilize indices such as the trade intensity index, trade complementarity index, and constant market share analysis to analyze further the bilateral trade flows and patterns and the trade potential of agriculture trade between India and China. It would also be beneficial to examine the impact of trade policies and regulations on agriculture trade using gravity models, simulation methods, and stakeholder analysis.

The study highlights that India and China share common strengths and weaknesses in the agriculture sector. Therefore, both countries can mutually benefit by exchanging best practices, innovations, and technologies. These include addressing trade imbalances, reducing trade costs, improving market access, promoting value addition, fostering innovation, and facilitating technology transfer. The prospects for further enhancing trade cooperation and integration in the agricultural sector are promising, given the unique strengths and opportunities of both India and China. The study has identified potential for trade diversification, enhanced cooperation, and collaboration between India and China. Consequently, the study put forward the following recommendations for both countries to leverage their comparative advantages while addressing comparative disadvantages.

### 5.2. Recommendations

1. Exploring the potential of trade complementarity and diversification in agriculture: India and China can enhance their agriculture sector productivity and trade by utilizing their distinct advantages in areas such as HS03 (fish and crustaceans) for India and HS33 (essential oils and resinoids) for China and addressing shared challenges like live animal production and food safety. Diversifying product offerings, specifically in HS13 (Lac and Gums) and HS35 (Albuminoidal Substances), can create new sources of income. Furthermore, investing in infrastructure for India and addressing trade barriers for China can facilitate trade. Thus, strategic complementarity, diversification, and tackling shared challenges can boost their bilateral agricultural trade standing.
2. Strengthen trade collaboration and enhance dialogue on Agriculture: With a particular focus on technology transfer, innovation, quality standards, and market access, India and China should consider exchanging their best practices and experiences in advancing and implementing new technologies, including biotechnology, precision agriculture, and digital agriculture, to enhance their production and competitive edge. Furthermore, both countries should consider proper negotiating and implementing preferential and free trade agreements, aiming to diminish or eradicate tariff and non-tariff obstacles within the agricultural sector to facilitate trade flows.
3. Improvement in domestic policies and agriculture sector reforms for India: As mentioned earlier in the study, China’s agriculture output per hectare is far greater than India’s. Therefore, in order to reduce its trade deficit with China and improve the overall performance of the agriculture sector, India needs to bring agriculture reforms, particularly in infrastructure, i.e., roads, railways, ports, storage facilities, cold chains, and logistics networks, to reduce transportation costs and post-harvest losses.). Additionally, it is imperative to attract domestic investment and FDI in the agricultural sector and rationalize subsidies and support programs for the agriculture sector to prevent market distortion and trade disputes. Moreover, it is essential to optimize the regulatory framework of the agriculture sector to reduce the administrative burden and create an attractive business environment.
4. Enhancing regional integration and agriculture cooperation through international organizations: India and China are members of multiple international organizations (i.e., the United Nations, the World Trade Organisation, BRICS, the G20, the Shanghai Cooperation Organisation, and China-Russia-India mechanisms) [31], and these organizations present a significant prospect for India and China to expand their market access, broaden trade diversification, and strengthen trade diversification in the agriculture sector with each other and other regional counterparts. Furthermore, these platforms allow both countries to address shared challenges and concerns in the agriculture sector, including but not limited to food security, climate change, environmental preservation, and rural development, for the overall growth of bilateral trade.

## Supporting information

S1 File (XLSX)
